# Digital Tools for Information, Communication, Support, and Family Engagement in Adult Intensive Care Units: A Scoping Review

**DOI:** 10.3390/healthcare14131944

**Published:** 2026-07-01

**Authors:** Vincenzo Bosco, Giuseppe Mazza, Rita Nocerino, Helenia Mastrangelo, Francesco Limonti, Eugenio Garofalo, Patrizia Doldo, Silvio Simeone, Federico Longhini, Giuseppe Neri, Caterina Mercuri

**Affiliations:** 1 Department of Medical and Surgical Sciences, University Hospital Mater Domini, Magna Graecia University, 88100 Catanzaro, Italygiuseppe.mazza@unicz.it (G.M.);; 2 Department of Biomedicine and Prevention, University of Rome “Tor Vergata”, 00133 Rome, Italy; 3 Department of Translational Medical Science, Federico II University Hospital, 80131 Naples, Italy; rita.nocerino@unina.it; 4 ASP Catanzaro, 88100 Catanzaro, Italy; 5 Clinical and Experimental Medicine Department, Magna Graecia University, 88100 Catanzaro, Italy

**Keywords:** intensive care unit, family-centered care, digital health, virtual visiting, scoping review

## Abstract

**Background:** Admission to an intensive care unit (ICU) exposes family members of adult patients to substantial informational, emotional, and decisional burden. In recent years, digital tools have increasingly been used to support communication, information delivery, virtual visiting, psychological support, diary writing, and surrogate decision making in ICU settings, although the available literature remains heterogeneous in terms of intervention type, purpose, timing, and outcomes assessed. **Methods:** A scoping review was conducted according to Joanna Briggs Institute methodology and reported following PRISMA-ScR. The literature search was performed between January and March 2026 in PubMed/MEDLINE, Scopus, and CINAHL. After duplicate removal, title/abstract screening, and full-text assessment, 32 studies were included in the qualitative synthesis. **Results:** The included studies were published between 2016 and 2026, used heterogeneous methodological designs, and originated from different international contexts. Six main categories of digital tools were identified: educational websites and online information resources; decision aids and tablet-based tools; virtual visiting and video communication systems; digital diaries and writing practices; psychological support or self-management applications; and digital assessment or family-engagement platforms. Overall, informational and communication-oriented tools appeared to provide the clearest signals of usefulness for family orientation, information access, communication, and relational continuity, whereas evidence regarding psychological and decisional outcomes remained more variable and largely preliminary. **Conclusions:** Digital tools for family members of adult ICU patients represent a relevant and evolving component of family-centered critical care. Their value appears to depend on the family need addressed, the timing of implementation, and their integration into clinical workflows. Overall, the available literature suggests that digital tools may be particularly useful for family orientation, information access, and communication, whereas their impact on psychological and decisional outcomes remains less certain and requires further investigation.

## 1. Introduction

Admission to an intensive care unit (ICU) is often sudden, unexpected, and emotionally overwhelming, not only for patients but also for their family members. Relatives of critically ill adults are frequently exposed to fear, uncertainty, informational needs, emotional distress, and a perceived loss of control during the ICU stay [1,2,3]. The experience of critical illness and the ICU environment may therefore represent a highly destabilizing event, contributing to psychological burden both during hospitalization and after discharge or bereavement [4].

In this context, the quality of communication between health care professionals and families is a central component of family-centered critical care. Clear, timely, and compassionate communication may improve relatives’ understanding of the patient’s condition, treatments, prognosis, and care trajectory [1]. It may also support their involvement in shared or surrogate decision making, particularly when decisions are complex and prognosis is uncertain [5,6]. This is especially relevant in adult ICU patients, who are often unable to communicate or take part in decision making because of their critical condition, sedation, mechanical ventilation, or impaired consciousness. In such circumstances, family members and surrogate decision-makers become key interlocutors in the care process [5,7].

From a nursing perspective, family communication and support are especially relevant, as ICU nurses are often involved in mediating interactions between patients, relatives, and the multidisciplinary team [8,9]. Nurses may also contribute to the implementation of family-facing digital interventions, particularly when these tools require preparation, guidance, or integration into ICU workflows [10,11]. Their role places them in a key position to facilitate understanding, continuity, emotional support, and family engagement throughout the ICU trajectory [8,9]. In recent years, digital technologies have increasingly entered this field as potential resources to support families of critically ill patients [1,2]. Rather than constituting a single type of intervention, these tools appear to operate across different functional domains [5,12].

A first domain includes informational and educational tools, such as ICU websites, brochure-linked websites, and online learning resources, designed to improve relatives’ understanding of the ICU environment, procedures, prognosis, and care processes [1,13]. Some studies also describe how families search for online information during ICU admission, suggesting that digital resources may complement, but not replace, professional communication [14,15].

A second domain includes communication and connection tools, such as videoconferencing systems, virtual visiting platforms, messaging-based communication solutions, and applications for communication with non-vocal patients. These tools aim to preserve family–patient contact and facilitate interaction with the ICU team when in-person communication is limited or insufficient [2,8]. Other communication-oriented tools, including app- or messaging-based systems, have also been explored in specific ICU contexts [16,17].

A third domain includes decision-support tools, such as web-based or tablet-based decision aids. These tools have been developed to prepare surrogate decision-makers for family meetings, clarify patient values, and support complex treatment decisions [5,6]. Additional studies suggest that the format and emotional demands of decision-support tools may influence family experience and cognitive burden [18,19].

A fourth domain includes psychological support and self-management interventions, particularly app-based programs intended to help family members manage distress, anxiety, depressive symptoms, or dimensions of post-intensive care syndrome-family (PICS-F) [20,21]. Evidence from specific ICU contexts also suggests that digitally mediated family education or communication may influence anxiety and satisfaction, although findings remain context-dependent [17,22].

Finally, a fifth domain includes digital diaries, writing-based tools, and follow-up or assessment platforms. Digital diaries and writing practices may support memory reconstruction, meaning-making, and family participation in the narration of the ICU experience [12,23]. Other digital assessment or follow-up platforms may extend family involvement across the ICU and post-ICU trajectory [24,25].

Existing evidence suggests that these categories may have different strengths and limitations. Informational tools appear to provide relatively clearer signals for orientation, access to information, and understanding [1,22]. Evidence regarding their impact on psychological outcomes remains more variable [3,22]. Communication tools, particularly virtual visiting and digital communication platforms, may help preserve family–patient connection and relational continuity [2,8]. Their usefulness appears to depend on implementation context, staff mediation, and organizational support [9,26]. Decision-support tools have generally been reported as acceptable or useful for preparation and values clarification [6,7]. However, their effects on prognostic concordance, decisional burden, and psychological distress appear less consistent [5,18]. Psychological support apps remain promising but preliminary, particularly for anxiety, depression, and PICS-F-related outcomes [20,21]. Digital diaries and writing-based tools may support memory reconstruction, meaning-making, and family engagement [12,23]. Their implementation in routine care appears closely linked to workflow integration and professional engagement [10,11].

However, despite this growing body of work, the literature remains fragmented. Studies differ substantially in design, population, intervention type, timing, implementation setting, and outcomes assessed.

Moreover, many publications focus on single categories of tools or on isolated aspects of family experience, such as information, communication, distress, or surrogate decision making [24,25]. As a result, the literature still offers only a partial view of how these interventions function within the broader organization of adult ICU care and family-centered practice. An additional source of heterogeneity may derive from the fact that tools with different functional purposes were often evaluated against different, and sometimes non-equivalent, outcomes. Even within the same functional domain, interventions are often heterogeneous and not directly comparable [24,25]. This heterogeneity includes simple informational websites, decision-support platforms, psychological applications, virtual visiting systems, digital diaries, and post-ICU e-health tools [5,12]. Some studies focus primarily on the acute ICU phase [1,2]. Others address post-ICU follow-up, psychological support, or hybrid interventions spanning recovery and longer-term trajectories [20,24]. App-based self-care and post-ICU outcome platforms further illustrate this temporal extension beyond the acute ICU stay [21,27]. As a result, it remains difficult to understand which types of digital tools are being used, which family needs they are intended to address, and how they may be relevant to family-centered critical care and nursing practice.

A scoping review is therefore appropriate to map the breadth of available evidence, clarify the main functional categories of digital tools described or evaluated for families of adult ICU patients, identify the needs they address, and summarize the outcomes most frequently explored [28,29]. Accordingly, the aim of this scoping review was to synthesize and organize the available evidence on digital tools used for information, support, communication, family engagement, and decision making among family members of adult ICU patients, with particular attention to their functional role, the family needs they address, and their relevance for family-centered critical care and nursing practice.

## 2. Materials and Methods

This literature review was conducted as a scoping review with the aim of broadly and systematically mapping the available evidence on digital tools used in adult intensive care unit (ICU) settings to inform, support, communicate with, involve, or connect family members of critically ill patients. A scoping review design was considered appropriate because of the expected heterogeneity of the available literature in terms of study design, type of digital tool, target population, timing of use, and intervention purpose. The review was conducted in accordance with the methodological guidance of the Joanna Briggs Institute and reported according to the PRISMA extension for Scoping Reviews (PRISMA-ScR) [28,29]. The review protocol was not prospectively registered.

### 2.1. Review Question

The review was guided by the following question, structured according to the Population–Concept–Context (PCC) framework recommended for scoping reviews [28]: Which digital tools have been described or evaluated to support information, communication, psychological support, family engagement, or decision making among family members of adult patients admitted to an intensive care unit?

The Population comprised family members, informal caregivers, relatives, significant others, and surrogate decision makers of adult ICU patients. The Concept encompassed any digital tool, including educational websites, virtual visiting platforms, decision aids, psychological support applications, digital diaries, and assessment or monitoring tools, developed or evaluated to address family-facing informational, communicative, psychological, or decisional needs. The Context was the adult intensive care setting; studies positioned along the ICU and post-ICU continuum were considered eligible when they provided evidence directly relevant to family-facing digital tools in the critical care trajectory.

### 2.2. Search Strategy

The literature search was conducted between January and March 2026 in three electronic databases: PubMed/MEDLINE, Scopus, and CINAHL. The search strategy was developed around three main conceptual domains: family members/caregivers, intensive care/critical care, and digital or online tools. The complete search strategy used in PubMed is reported in Supplementary Table S2. The strategy was subsequently adapted to the syntax of Scopus and CINAHL while preserving the same conceptual structure and semantic meaning across databases.

To increase the relevance of the search results, the following filters were applied in PubMed: last 10 years, Humans, and Adult: 19+ years. Overall, 5104 records were identified, including 1120 from PubMed/MEDLINE, 2935 from Scopus, and 1049 from CINAHL. The CINAHL search did not identify additional eligible studies beyond those already retrieved through PubMed/MEDLINE and Scopus.

### 2.3. Eligibility Criteria

Studies were considered eligible if they referred to adult patients admitted to ICU and their family members, informal caregivers, relatives, significant others, or surrogate decision-makers. Studies were included if they described, developed, tested, implemented, or evaluated digital, web-based, mobile, tablet-based, videoconferencing-based, or online tools used for family-facing information, psychological support, communication, family engagement, virtual visiting, decision making, diary writing, assessment, or monitoring.

Consistent with the exploratory nature of a scoping review, studies positioned along the ICU/post-ICU continuum or involving mixed populations were also considered eligible if they included a substantial family component or provided information directly relevant to the development, understanding, implementation, or evaluation of family-facing digital tools.

Studies were excluded if they were exclusively pediatric or neonatal, focused only on patients without a substantial family component, addressed digital tools unrelated to the ICU context, or used digital technology solely as a technical means for data collection or simulation rather than as the intervention, resource, or tool of interest for family members.

### 2.4. Study Selection

All identified records were exported and managed using Rayyan. After duplicate removal, the selection process was conducted in two sequential phases: title and abstract screening, followed by full-text assessment. Screening was performed independently by two reviewers, who assessed the relevance of each record against the review question and predefined eligibility criteria. Disagreements were resolved through discussion, and, when necessary, a third reviewer made the final decision.

**Figure 1:**
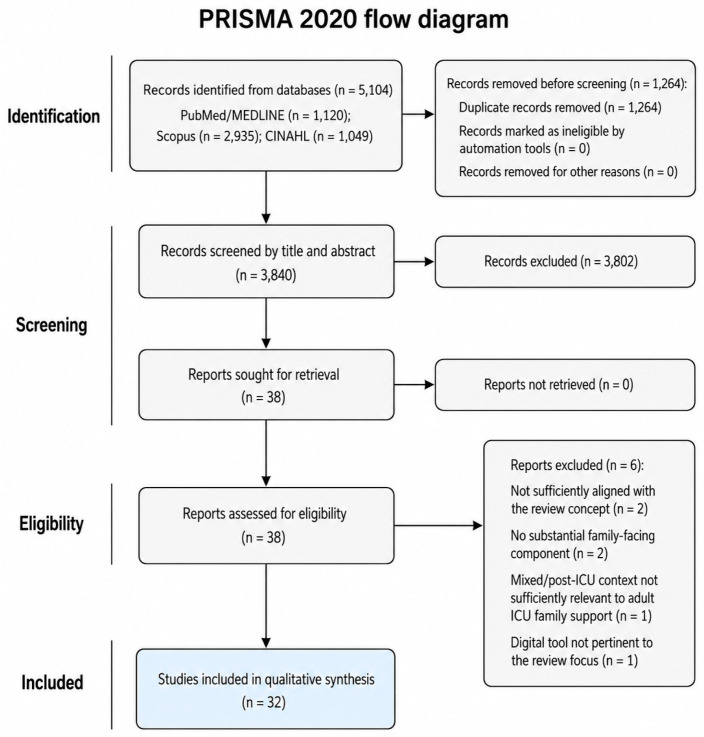
PRISMA 2020 flow diagram of study selection, including records removed before screening and aggregated reasons for full-text exclusion.

After removal of 1264 duplicates, 3840 records were screened by title and abstract. No records were marked as ineligible by automation tools or removed for other reasons before screening. Of these, 3802 records were excluded because they were not relevant to the review question, did not refer to the adult ICU context, focused exclusively on patients, or did not include a genuine digital tool directed toward family members. Thirty-eight full-text reports were retrieved and assessed for eligibility. All reports sought for retrieval were obtained. During full-text assessment, six studies were excluded because they did not sufficiently address the review concept, target population, or adult ICU context. At the end of the selection process, 32 studies were included in the qualitative synthesis. The identification, screening, eligibility, and inclusion process is reported in the PRISMA flow diagram (Figure 1).

### 2.5. Data Charting

For each included study, a data charting table was developed to collect the main methodological, descriptive, and content-related characteristics relevant to the review’s aim. Extracted information included author and year, database source, title, study design, sample, setting and population, type of digital tool, study aim, main outcomes, main findings, and rationale for inclusion. The complete data extraction table is reported in Supplementary Table S1.

Data charting was performed independently by two reviewers using a predefined charting form developed for this review. The form was piloted on a subset of included studies and refined before full data extraction. Any discrepancies were resolved through discussion, with involvement of a third reviewer when needed.

### 2.6. Data Synthesis

Given the marked heterogeneity of study designs, populations, digital technologies, intervention aims, and outcomes assessed, no meta-analysis was performed. Findings were synthesized narratively and thematically. The synthesis aimed to identify the main types of digital tools described in the literature, the family needs addressed by these tools, and the principal outcomes reported. The thematic categories were developed through an iterative process of comparative reading across the included studies. In particular, studies were grouped according to the primary function of the digital tool, the main family need addressed, and the context in which the intervention was used or evaluated. Category boundaries were discussed among the reviewers and refined until consensus was reached, with the aim of producing functionally meaningful groups that could support narrative synthesis across a heterogeneous evidence base. This categorization was intended as an analytic aid to interpretation rather than as a rigid classification of mutually exclusive intervention types.

Studies were organized into recurring thematic categories: online information tools and educational websites; decision aids and tablet-based tools; virtual visiting and video communication systems; digital diaries and writing practices; psychological support or self-management applications; and digital assessment or family-engagement platforms.

### 2.7. Critical Appraisal

A formal critical appraisal of the methodological quality of the included studies was not performed. This decision was consistent with the main purpose of the scoping review, which was to map the extent, range, and characteristics of the available literature rather than to determine intervention effectiveness or produce a quality-weighted synthesis. This approach is consistent with established scoping review methodology [28,29]. It is also aligned with guidance suggesting that formal quality appraisal is not always required when the aim is to map the extent and characteristics of a body of evidence rather than to produce an effectiveness synthesis [30,31].

## 3. Results

### 3.1. Study Selection

The literature search conducted in PubMed/MEDLINE, Scopus, and CINAHL identified a total of 5104 records: 1120 from PubMed/MEDLINE, 2935 from Scopus, and 1049 from CINAHL. After the removal of 1264 duplicates, 3840 records were screened by title and abstract. Of these, 3802 were excluded because they were not relevant to the review question, did not refer to adult ICU settings, focused exclusively on patients, or did not include a genuine digital tool directed toward family members.

Thirty-eight full-text reports were assessed for eligibility, and all were retrieved. During full-text assessment, six studies were excluded because they did not sufficiently address the review concept, target population, or adult ICU context. Reasons for full-text exclusion included insufficient alignment with the review concept, absence of a substantial family-facing component, limited relevance of mixed or post-ICU contexts to adult ICU family support, and digital tools not pertinent to the review focus.

These exclusion categories were applied during full-text assessment to distinguish studies that mentioned ICU, families, or digital technologies only marginally from studies in which the digital tool was directly relevant to family-facing information, communication, support, engagement, decision making, diary writing, assessment, or monitoring in adult ICU care. Reports were excluded when the digital component was used only as a technical means for data collection, when the family component was absent or incidental, or when the setting and population were not sufficiently connected to adult ICU family support.

The final corpus consisted of 32 studies included in the qualitative synthesis.

### 3.2. General Characteristics of Included Studies

The included studies were published between 2016 and 2026, with a higher concentration in recent years, reflecting increasing interest in digital tools to support family members of adult ICU patients. The final corpus included 26 studies identified through PubMed/MEDLINE and six additional eligible studies identified through Scopus. The CINAHL search did not identify further eligible studies beyond those already captured in the other databases. The methodological and descriptive characteristics of the 32 included studies are reported in Supplementary Table S1. Supplementary Table S1 also distinguishes studies according to their primary evidence perspective (family-level, professional-facing, mixed, or contextual/resource-based) to improve transparency regarding the source of reported data.

The methodological distribution of the included studies according to primary study design is summarized in Supplementary Table S3.

Overall, the included studies were methodologically heterogeneous and comprised qualitative studies, observational studies, multicentre surveys, usability studies, pilot studies, randomized trials, implementation evaluations, and user-centered development studies. This heterogeneity reflects the evolving nature of the field, in which tested digital interventions coexist with feasibility studies, early-stage prototypes, implementation evaluations, and studies exploring family information or communication needs.

The studies originated from several international contexts, including the United States, Italy, France, the United Kingdom, Brazil, the Netherlands, China, Ireland, Australia, Austria, Switzerland, Denmark, and Iran. This geographical distribution suggests that interest in digital family support in ICU is not limited to a single health care system [1,2]. It also reflects the growing integration of digital tools into family-centered critical care pathways across different clinical contexts [8,9].

The digital tools described across the included studies were highly diverse. They included educational websites, brochure-linked online resources, and online information tools [1,15]. Other studies focused on web-based decision aids, tablet-based tools, and applications for communication with non-vocal patients [5,16]. Further interventions included psychological support apps, virtual visiting systems, social media or messaging-based communication platforms, digital diaries, online assessment tools, and digital models for family involvement in early rehabilitation [8,12,32].

The thematic categories of the included studies are summarized in Table 1.

The findings are presented below according to these thematic categories, with attention to study design, main family needs addressed, and reported outcomes.

### 3.3. Thematic Synthesis

#### 3.3.1. Online Information Tools and Educational Websites

This category included methodologically diverse studies. These comprised interventional or observational studies evaluating family-facing informational tools [1,22]. Other studies examined social media-based education or online information-seeking during ICU admission [14,33]. Additional resource-mapping, usability, and randomized studies explored the quality, usability, or psychological impact of online informational resources for relatives [3,13,34]. Overall, these studies addressed families’ need for orientation, access to information, and understanding of the ICU environment and care processes.

Across these studies, informational and educational tools were generally associated with better understanding of prognosis, procedures, and ICU processes [1,22]. In one context, social media-based education was also associated with higher satisfaction and lower anxiety among family members [33]. At the same time, observational and survey-based evidence showed that family members and surrogate decision-makers frequently search for information online during an ICU admission, although ICU physicians and nurses remain the most trusted and influential sources of clinical information [14,15]. Other studies highlighted the variability and uneven quality of informational resources available in ICU settings, supporting the need for more structured and accessible digital educational pathways for families [13,34]. A descriptive multicentre study conducted in Spanish ICUs also highlighted gaps in the informational content provided to relatives through ICU admission brochures, particularly regarding nursing information, suggesting that formal family-facing information resources may remain incomplete or inconsistent across settings [35]. However, evidence from a multicentre randomized trial suggested that, although online information tools are acceptable and used by families, their effects on more complex psychological outcomes such as post-traumatic stress symptoms may be limited or inconsistent [3]. Taken together, these findings suggest that online informational tools may be particularly useful for family orientation and understanding [1,22]. Their impact on broader psychological outcomes, however, remains uncertain [3,33].

#### 3.3.2. Decision Aids and Tablet-Based Tools for Communication and Decision Making

This group comprised a qualitative acceptability study [36], a user-centered development and pilot usability study [6], a pilot randomized trial [7], a multicentre randomized clinical trial [5], a comparative study on cognitive load [18], and a pilot intervention focused on emotion regulation [19]. Collectively, these studies addressed the needs of surrogate decision-makers for preparation, values clarification, emotional support, and more structured participation in ICU decision making.

Across this category, digital decision-support tools were generally found to be acceptable and feasible [6,7]. Qualitative evidence also suggested that such tools may help family members prepare for meetings with clinicians, clarify patient values, and navigate complex treatment decisions [36]. However, their impact on distal or high-stakes outcomes was less consistent. In the largest multicentre randomized trial, a personalized web-based decision aid did not improve prognostic concordance or clearly reduce psychological distress, although it was associated with lower decisional conflict [5]. Other studies suggested that the way information is digitally presented may matter: for example, different formats of electronic decision aids appeared to generate different levels of cognitive load among family members [18]. More recent pilot work has also explored emotion-regulation components, with preliminary signals suggesting potential benefits for depressive symptoms, although within early-stage designs [19]. Overall, the evidence suggests that decision-support tools may be valuable for process-related outcomes such as preparation, usability, and decisional support [6,7]. Stronger evidence is still needed regarding their effects on clinical, psychological, and decisional endpoints [5,19].

#### 3.3.3. Virtual Visiting and Video Communication

The literature on virtual visiting and digital communication included a qualitative descriptive study on barriers and facilitators [8], a multicentre prospective observational cohort study [2], a qualitative descriptive study focused on ethical issues [37], and more recent implementation and user-experience studies from Denmark and Ireland [9,26]. Together, these studies addressed the need for connection, presence, and relational continuity between patients and families when physical visitation was limited or constrained.

In addition, a mixed-methods pilot study on the VidaTalk™ communication application suggested that app-based communication support may help families of non-vocal ICU patients maintain interaction, reduce frustration, and improve the quality of communication when direct verbal exchange is not possible [16].

A comparative study conducted in a postoperative intensive care setting also suggested that a WeChat-based communication model may support family communication, improve satisfaction, and facilitate relational continuity through messaging-based interaction [17].

Across this group, virtual visiting was consistently described as a potentially important means of maintaining family–patient contact and reducing the sense of separation associated with critical illness and ICU admission [2,8]. At the same time, the family experience of virtual visiting was shown to be highly dependent on contextual and relational factors, including preparation, timing, ease of use, staff presence, and the emotional management of the interaction [8]. Observational evidence suggested that family members engaging in virtual visiting may experience high levels of distress, anxiety, and depression, although distress appeared to decrease after one or more digital visits [2]. The literature also emphasized that virtual visiting raises ethical issues, including privacy, dignity, emotional burden, and equity of access [37]. Its sustainability in routine ICU care appears to require organizational integration and professional mediation [9,26]. Overall, these findings suggest that virtual visiting should be understood not simply as a technical substitute for in-person visits, but as a relational intervention that requires preparation and staff mediation [8,37]. Its quality and sustainability also appear to depend on implementation context and organizational support [9,26].

#### 3.3.4. Psychological Support and Self-Management Applications

This category included two family-targeted app-based studies by Petrinec and colleagues—a feasibility study [20] and a randomized pilot study [21]—as well as one randomized trial of a family-participatory rehabilitation model supported by internet and mobile technology [32]. These studies addressed the need for emotional support, coping, symptom management, and, in one case, more active family participation in the care pathway.

The available evidence suggests that mobile or app-based interventions may represent a feasible way of delivering psychological support to family members at risk of PICS-F [20,21]. Early findings also suggest that such tools may support self-care and may be associated with reductions in anxiety and depressive symptoms, although the current evidence remains preliminary and is limited by pilot designs and relatively small samples [20,21]. In parallel, the study on digital family-participatory rehabilitation suggested that internet-supported interventions may also extend beyond emotional support, enabling family involvement in recovery-oriented care processes [32]. Taken together, these studies suggest that digital tools may support coping and emotional regulation among family members, although this evidence remains preliminary [20,21]. Digital interventions may also contribute to participatory models of care, but this area requires further confirmation in larger and more comparable studies [32].

#### 3.3.5. Digital Diaries, Writing Practices, and Family-Facing Narrative Tools

This group included a pilot usability study from the relatives’ perspective [12], a service innovation and implementation evaluation of an ICU e-diary [10], a professional survey on determinants of digital diary implementation [11], and a qualitative study on family writing practices during ICU admission and early bereavement [23]. These studies addressed needs related to meaning-making, continuity, narrative reconstruction, family engagement, and humanization of care.

Across these studies, digital diaries and writing-based tools were generally perceived as useful and acceptable, particularly when they facilitated collaborative contributions from family members and health care professionals [10,12]. Implementation-focused evidence suggested that accessibility, education, local champions, and professional understanding of the tool’s value may be key determinants of successful integration into clinical practice [10,11]. At the same time, qualitative findings indicated that the value of writing practices may lie not only in later rereading, but also in the act of writing itself as a means of processing the ICU experience and constructing meaning [23]. Overall, this category suggests that narrative digital tools may contribute to continuity, memory, and family involvement in the ICU experience [12,23]. Their contribution to humanization and routine clinical use appears closely linked to workflow integration and staff engagement [10,11].

#### 3.3.6. Digital Assessment Tools and E-Health Platforms

This final category included a cross-cultural validation study of an online family-administered delirium assessment tool [25], a cross-sectional member consultation on priorities for a post-ICU e-health platform [24], and a usability development and evaluation study of an electronic patient-reported outcome system that included family members and caregivers [27]. These studies addressed needs related to assessment, monitoring, follow-up continuity, and the extension of family involvement beyond the acute ICU phase.

Although fewer in number, these studies suggest that digital tools may also support family participation in selected assessment tasks during ICU care [25]. Other studies suggest that digital platforms may contribute to post-ICU follow-up and outcome-monitoring processes involving families [24,27]. The online adaptation of the Family Confusion Assessment Method indicated that digitally mediated family involvement may be feasible in specific assessment tasks such as delirium detection during online visits [25]. In parallel, studies on e-health platforms and post-ICU electronic outcome systems pointed to family needs related to accessibility, personalization, continuity of support, and longer-term follow-up [24,27]. Overall, this group broadens the scope of digital family support beyond the acute ICU stay [24,27]. It also highlights the possible role of digitally mediated family involvement in selected assessment tasks during ICU care [25].

A structured summary of studies reporting null, limited, or mixed effects is provided in Supplementary Table S4.

## 4. Discussion

Taken together, the 32 included studies depict a rapidly expanding but still methodologically and conceptually heterogeneous field. The digital tools described in the literature do not constitute a single homogeneous category, but rather a broad set of interventions developed to address different family needs, including understanding the ICU environment, accessing reliable information, preparing for surrogate decision making, maintaining contact with the patient, managing emotional distress, participating in narrative reconstruction, and supporting recovery or follow-up. Although many tools were reported as acceptable, feasible, and perceived as useful, their overall effectiveness remains difficult to synthesize because of heterogeneity in study design, timing, implementation, and outcomes assessed. A recurring pattern was that tools primarily designed for information delivery and communication appeared to show more consistent benefits than tools aimed at modifying complex psychological or decisional outcomes.

A first key consideration concerns the relationship between the function of the tool and the type of outcome assessed. Tools primarily designed to provide information or orientation tended to show more consistent benefits on understanding, access to information, and satisfaction [1,22]. Usability and survey-based studies further suggest that these tools may be useful when they complement verbal communication and can be revisited over time [13,15]. By contrast, tools aimed at broader psychological outcomes, such as anxiety, depression, or post-traumatic stress symptoms, showed more variable results [3,21]. These outcomes are likely influenced by multiple emotional, clinical, relational, and contextual factors, and may therefore be less responsive to digital resources alone.

A second relevant issue is timing across the care trajectory. Some tools appear particularly suited to the acute ICU phase, when families need orientation, information, and communication support [1,8]. Digital diaries and writing-based tools may become more meaningful later, when relatives attempt to reconstruct events and integrate the ICU experience into a broader narrative [12,23]. App-based psychological support may also be more relevant when distress persists beyond the immediate crisis and family members have sufficient emotional space to engage with self-management content [20,21]. This temporal dimension may partly explain why findings differed across tool categories and outcomes.

The findings on decision-support tools highlight the limits of a purely informational approach to surrogate decision making. These tools were generally acceptable and often perceived as useful, but they did not consistently improve prognostic concordance or reduce psychological burden [5,7]. The way information is presented may also matter, as different electronic decision-aid formats appeared to generate different levels of cognitive load [18]. This suggests that decision making in ICU is not only cognitive but also relational and emotional, shaped by uncertainty, fear, fatigue, and evolving clinical conditions. Decision aids may reduce decisional conflict, but they may still be insufficient to align understanding between families and clinicians or to reduce distress when emotional burden remains high [5]. Recent pilot work on emotion regulation is therefore relevant, but remains preliminary [19].

A similar pattern emerges in relation to virtual visiting and digital communication tools. Their value appears to depend less on the technology itself than on their ability to preserve relational continuity under constrained circumstances [2,8]. What matters is not merely whether a virtual encounter takes place, but how it is prepared, mediated, and embedded in ICU practice. The findings suggest that digital communication may be more useful when it is supported by staff presence, appropriate timing, and family preparation [8,9]. Its sustainability also appears to depend on organizational support and integration into routine ICU workflows [9,26]. When these conditions are absent, the same technologies may become distressing, confusing, or ethically problematic [8,37]. This helps explain why studies in this area often report both benefit and burden: digital communication may reduce separation, but it may also expose families to emotionally intense encounters without adequate support [2,8,37].

This area also brings privacy and confidentiality into sharper focus. Virtual visits and messaging-based communication may involve the circulation of sensitive clinical and emotional content through technological platforms [17,37]. Digital diaries may add further privacy considerations because family- and staff-generated entries can become part of a shared narrative around highly vulnerable patients [10,12]. Privacy should therefore be considered a central ethical issue rather than a secondary technical concern [37]. From a clinical and nursing perspective, family-facing digital tools require explicit governance, professional guidance, and clear boundaries around consent, documentation, and communication practices [10,11]. Implementation studies further suggest that these safeguards need to be integrated into local workflows rather than treated as optional technical precautions [11,26].

A further issue concerns the conditions under which digital tools can be meaningfully implemented in family-centered ICU care [10,37]. Across the included studies, the impact of these interventions appeared to depend not only on the intrinsic features of the tool, but also on organizational readiness, staff mediation, and workflow integration [9,11]. Timing also seemed to matter, particularly when tools were introduced during periods of acute family distress or high decisional burden [20,21]. These findings suggest that digital tools do not operate as neutral add-ons, but as context-dependent interventions whose usefulness may vary according to implementation conditions and local resources [10,26]. Digital inequalities and digital literacy are also likely to be relevant [8]. Family members may differ in access to devices, internet connectivity, confidence in using digital platforms, and emotional readiness to engage with digital content during crisis [8,9]. These differences may help explain why some interventions appear feasible and acceptable in one context but more difficult to implement or less beneficial in another [26]. From a practical perspective, successful implementation may require not only technological availability, but also staff training, clear guidance, family support, and attention to equity in access and usability [9,11].

The findings on digital diaries and writing practices are important because they point to a dimension of ICU care that is often less visible in outcome-driven research: narrative reconstruction of the experience [12,23]. Their contribution appears to lie not only in documentation, but also in helping families create continuity across an experience that is often fragmented and difficult to interpret retrospectively. This may explain why these tools are linked to humanization, family engagement, and relational continuity [10,12]. Implementation evidence also suggests that their routine use depends on workflow integration and professional engagement [10,11]. At the same time, their benefits likely depend on how actively families and professionals engage with them, and on whether writing is integrated into routine care rather than treated as an optional add-on [10,11].

The inclusion of studies on psychological support apps suggests a possible shift from digital tools as passive resources toward digital tools as active supports for coping [20,21]. Digitally supported family participation in care may represent a related, but still distinct, direction of development [32]. This broadens the role of family members from recipients of information to contributors to selected components of care or recovery. However, this area remains at an early stage, and its promise should be interpreted cautiously. Feasibility and acceptability are important, but they are not sufficient indicators of clinical impact, and future studies should clarify which family subgroups benefit most, under what conditions, and at what time points [20,21,32].

Overall, the main contrast in the literature may not be between “effective” and “ineffective” tools, but between tools whose functional purpose matches the family need and phase of care, and tools whose outcomes are assessed at a level that exceeds what the intervention is realistically designed to change. This perspective may help explain part of the inconsistency across studies. In several cases, the issue may be less the failure of the intervention itself than a mismatch between the tool, its timing, its mechanism of action, and the outcome selected for evaluation.

Finally, this remains a developing field. Many included studies were pilot, feasibility, usability, or user-centered development studies, indicating that the literature is still partly concerned with defining and refining these tools before stronger comparative evidence can be generated [6,7]. Other studies focused primarily on usability, implementation, or early-stage testing rather than on confirmatory effectiveness [12,13]. Future research should therefore move toward clearer functional distinctions between interventions and stronger attention to timing across the ICU and post-ICU continuum [5,20]. It should also develop more explicit privacy and implementation frameworks, with outcome selection better aligned with the actual purpose of each tool [10,11].

### 4.1. Implications for Nursing Practice

The findings of this review have direct implications for nursing practice in adult ICU settings. ICU nurses are often central to family communication, education, emotional support, and bedside coordination. Therefore, digital tools should not be viewed as external technological add-ons, but as potential nurse-sensitive strategies to support family-centered care.

First, educational websites and online information resources may help nurses provide families with consistent, accessible, and repeated information about the ICU environment, procedures, devices, and care pathways. These tools may be particularly useful when family members feel overwhelmed during verbal communication or need to revisit information after clinical updates.

Second, virtual visiting and digital communication platforms require nursing involvement in preparation, timing, privacy protection, emotional containment, and support during or after the interaction. Without professional mediation, these tools may expose families to distressing images or ambiguous information without adequate explanation.

Third, digital diaries and writing tools may support the humanization of care and family engagement, but their implementation requires clear workflows, staff education, and shared responsibility within the ICU team. Nurses may have a key role in inviting family participation, explaining the purpose of the diary, and contributing to entries when appropriate.

Fourth, psychological support apps and decision-support tools may complement, but not replace, relational support from health care professionals. Nurses should be aware of their potential benefits and limitations, and should help identify family members who may need additional psychological, informational, or decision-making support.

Finally, implementation is crucial. The usefulness of a digital tool appears to depend not only on its content, but also on how it is introduced into the clinical environment [8,9]. Accessibility, staff training, local champions, and clarity of purpose may facilitate or hinder implementation [10,11]. Family usability and flexibility of access should also be considered when these tools are introduced into routine ICU care [9,26]. In this sense, digital tools in ICU may be more useful when they are embedded within a broader family-centered care model rather than simply added to existing practice [1,6].

### 4.2. Limitations

Several limitations of this review should be acknowledged. The included literature was highly heterogeneous in terms of study design, population, intervention type, timing, implementation setting, and outcomes assessed. While this heterogeneity was itself a rationale for adopting a scoping review approach, it constrained the possibility of direct cross-tool comparison and reduced the strength of cross-study interpretation [28,29]. A substantial proportion of the included studies consisted of pilot, feasibility, usability, acceptability, or user-centred development studies, rather than large confirmatory trials. Accordingly, much of the available evidence should be interpreted as preliminary signals of potential benefit rather than as definitive demonstrations of effectiveness. The search strategy was restricted to PubMed/MEDLINE, Scopus, and CINAHL, and did not encompass PsycINFO or grey literature sources, which may have limited the identification of studies addressing family mental health, emotional support, and the psychological dimensions of digital interventions. Furthermore, the use of automatic PubMed filters (last 10 years, Humans, Adult: 19+ years) may have reduced search sensitivity. As an indirect indication, five of the six studies retrieved exclusively through Scopus were identifiable in PubMed by their PMID, suggesting that they may have been present in the database but not captured by the filtered query. This circumstance does not affect the integrity of the final corpus, since all studies were identified through multi-database triangulation, but it indicates that the PubMed component alone may have underperformed relative to its potential yield. Restricting the search to publications from the last ten years, while intended to capture contemporary digital tools, may have excluded earlier foundational studies relevant to the development of this field. Publication bias cannot be excluded, as the review relied exclusively on indexed published literature and did not incorporate grey literature, local implementation reports, or unpublished studies. The corpus included studies positioned at the margins of the strictly defined focus, such as post-ICU interventions or mixed-population designs. Although this choice was deliberate and allowed a broader mapping of the phenomenon, it may have introduced some reduction in thematic homogeneity. The rapid pace of technological evolution in this field also means that some tools described in the included studies may already have changed in format, implementation context, or clinical relevance by the time of publication. No formal critical appraisal of methodological quality was performed, consistent with the purpose of a scoping review, which aims to map the extent and variety of available evidence rather than to produce an effectiveness synthesis. This approach, however, limits the inferential strength of the conclusions [28,29]. This limitation should be considered when interpreting findings across heterogeneous and preliminary bodies of evidence [30,31]. Finally, some included studies originated from the authors’ own research group or closely related lines of investigation [38,39]. Although study selection, data charting, and synthesis were conducted according to predefined procedures and independent review processes, this circumstance should be considered when interpreting the categorization and narrative synthesis of the evidence.

## 5. Conclusions

This scoping review shows that digital tools for family members of adult ICU patients represent a complex and rapidly evolving field in which informational, communicative, relational, decisional, and psychological support functions converge. Rather than constituting a single category of interventions, digital tools comprise a range of solutions designed to address specific family needs across the trajectory of critical illness, from understanding the ICU environment and maintaining connection with the patient to supporting decision making, participating in care, and processing the ICU experience.

Overall, the included studies suggest that many digital tools were reported as acceptable, feasible, and perceived as useful, particularly when they were designed to improve access to information, facilitate communication, or support connection between patients, families, and health care professionals. Their effectiveness on more complex psychological or decisional outcomes remains less certain, with findings that are promising but not yet homogeneous or definitive. Across the included literature, informational and communication-oriented tools appeared to provide the clearest signals of usefulness, particularly for orientation, access to information, and relational continuity, whereas evidence for psychological and decision-support outcomes remained more variable and largely preliminary.

A recurring finding is that the value of digital tools does not depend only on the technology itself, but on how the tool is designed, introduced, mediated, and integrated within family-centered care. Accessibility, ease of use, clarity of purpose, staff preparation, family support, and alignment with real ICU needs appear essential for these tools to produce meaningful benefits.

From a nursing perspective, digital tools may support family education, communication, emotional support, virtual presence, diary-based engagement, and participation in care. However, they should complement rather than replace relational and professional support from ICU teams. Future studies should use more robust and comparable designs and should distinguish more clearly between tools intended to inform, tools intended to support emotional needs, and tools designed to facilitate decisional, relational, or participatory processes.

In summary, digital tools may offer relevant opportunities to support the experience of family members in adult ICU settings, but their potential cannot be reduced to technical innovation alone. The central challenge is to understand which tools appear most appropriate for specific family needs, at which phase of the care pathway, and with what degree of integration into everyday nursing and multidisciplinary ICU practice.

## Figures and Tables

**Table 1:** Thematic categories of digital tools identified in the included studies.

Category	Examples of Tools	Main Family Need Addressed	Nursing Relevance
Online information tools and educational websites	ICU websites; online learning resources; brochure-linked websites	Orientation, understanding, information access	Supports repeated, accessible education and consistent explanations
Decision aids and tablet-based tools	Web-based decision aids; tablet communication tools; emotion-regulation apps	Surrogate decision making, values clarification, preparation for meetings	Complements nurse-mediated communication and emotional support
Virtual visiting and video communication	Video visits; bespoke ICU family-link systems; messaging platforms	Connection, presence, relational continuity	Requires preparation, privacy protection, timing, and emotional containment
Psychological support and self-management apps	Mobile CBT-based apps; self-care mental health apps	Anxiety, depression, PICS-F, coping	May help nurses identify and signpost families needing additional support
Digital diaries and writing practices	ICU e-diaries; web-based diaries; collaborative writing tools	Narration, memory, meaning-making, humanization	Facilitates family engagement and shared documentation of the ICU experience
Digital assessment and e-health platforms	Online FAM-CAM; e-health follow-up platforms; electronic PRO systems	Assessment, monitoring, follow-up continuity	Supports family involvement across ICU and post-ICU pathways

## Data Availability

No new data were created or analyzed in this study.

## Supplementary Materials

The following supporting information can be downloaded at: https://www.mdpi.com/article/10.3390/healthcare14131944/s1, Table S1. Methodological and descriptive characteristics of included studies; Table S2. Complete PubMed/MEDLINE search strategy; Table S3. Methodological distribution of the 32 included studies; Table S4. Studies reporting limited, null, or mixed effects of digital tools on family-related outcomes.

## Abbreviations

CBT	cognitive behavioural therapy
ICU	intensive care unit
JBI	Joanna Briggs Institute
PICS-F	post-intensive care syndrome-family
PRISMA-ScR	Preferred Reporting Items for Systematic Reviews and Meta-Analyses extension for Scoping Reviews
PRO	patient-reported outcome
