# Supplementary material for: Digital Tools for Information, Communication, Support, and Family Engagement in Adult Intensive Care Units: A Scoping Review
Source: Healthcare (Basel). 2026 Jul 1;14(13):1944. doi: 10.3390/healthcare14131944 (PMC13361844; doi:10.3390/healthcare14131944)
Supplement: Supplementary file 1 [file healthcare-14-01944-s001.zip › healthcare-4356687-supplementary.pdf]

Table S1. Methodological and descriptive characteristics of included studies

**Author_year**	**Database_source**	**Title**	**Design**	**Sample**	**Setting_population**	Primary evidence perspective	**Digital_tool**	**Aim**	**Main_outcomes**	**Main_findings**	**Why_included**	**Main_limitations**
**Cox et al., 2016 [27]**	PubMed	Usability Testing of an Electronic Patient-Reported Outcome System for Survivors of Critical Illness	Usability development and evaluation study	60 ICU survivors, family members, and PRO/programming experts	Recently ill ICU survivors and their families within an ongoing post-ICU clinical trial	Mixed	ePROSPER web-based electronic patient-reported outcome system	Develop and evaluate the usability of an electronic PRO system for ICU survivors and families	Usability scores; task completion time; error rates; user feedback	Usability improved across testing cycles (median score 40 to 73 to 95); all users completed ePROSPER within 20 minutes, 87% preferred it to paper, and the platform was implemented in a clinical trial without data errors.	Broader post-ICU digital monitoring/resource study involving families; included for completeness of the digital-family continuum.	Usability-focused study with a modest mixed sample of survivors and family members; the post-ICU setting and trial-specific context may limit transferability to family-facing tools used during ICU admission.
**Ernecoff et al., 2016 [36]**	PubMed	Key stakeholders' perceptions of the acceptability and usefulness of a tablet-based tool to improve communication and shared decision making in ICUs	Qualitative interview study	58 stakeholders (30 surrogates, 28 ICU care providers)	Adult ICU surrogate decision makers and ICU providers	Mixed	Proposed tablet-based communication and shared decision-making tool	Assess acceptability, usefulness, and design suggestions for a tablet-based ICU communication tool	Perceived acceptability and usefulness; desired content and design features	95% found the proposed tool acceptable and 98% endorsed at least 6 of 7 content domains; participants emphasized flexibility, interactivity, simple interface, and use as a supplement to—not replacement for—clinician communication.	Foundational family-facing digital communication/support study that informed later tablet/web interventions.	Qualitative perception study of a proposed tool; it did not assess clinical effectiveness or real-world implementation, and transferability should therefore be considered with caution.

**Mistraletti et al., 2017 [1]**	PubMed	A family information brochure and dedicated website to improve the ICU experience for patients' relatives: an Italian multicenter before-and-after study	Prospective multicenter before-and-after study	551 relatives received questionnaires; 332 responded overall. Phase-specific respondent numbers reported in the original article were 144 before and 179 after implementation.	Adult ICU relatives/family members	Family-level	Information brochure plus dedicated ICU website (www.intensiva.it)	Assess whether brochure + website improved family understanding and psychological outcomes without increasing staff workload	Understanding of prognosis/procedures; family satisfaction; anxiety, depression, post-traumatic stress symptoms	Intervention improved correct understanding of prognosis (69% to 84%) and therapeutic procedures (17% to 28%); after implementation 73% read the brochure and 19% viewed the website; post-traumatic stress symptoms appeared reduced, while website uptake remained modest.	Evaluates a family-facing website in adult ICUs with family-centered outcomes	Nonrandomized before-after design with possible temporal and responder bias; the relatively low website uptake may also have limited the magnitude of observable effects. Some reported denominators varied across questionnaire-based analyses.
**Nguyen et al., 2017 [14]**	PubMed	"RéaNet", the Internet utilization among surrogates of critically ill patients with sepsis	Prospective observational multicenter study	169 surrogates of 146 septic ICU patients	Adult ICU surrogates/proxies of critically ill patients with severe sepsis or septic shock	Family-level	General health-related Internet use; ICU website availability	Describe prevalence, characteristics, and predictors of Internet use among surrogates of critically ill septic patients	Internet use about sepsis; perceived reliability/usefulness of online information; predictors of use	55% of Internet users searched online about sepsis; most judged online information reliable/suitable/concordant; prior health-Internet use, nursing presence at family-physician meetings, younger patient age, and renal replacement therapy were associated with Internet use; 52% wanted a list of recommended websites.	Directly examines family/surrogate online information-seeking during adult ICU stay	Observational study based on self-reported Internet use; the focus on surrogates of septic patients may reduce generalizability to other ICU populations.

**Schnock et al., 2017 [34]**	PubMed	Identifying Information Resources for Patients in the Intensive Care Unit and Their Families	Multicenter content collection and gap analysis	Educational content/resources across 4 hospitals	ICU patients and family education resources	Contextual/resource-based	Conceptual digital learning center / information pathway (resource-mapping study)	Identify ICU educational content, develop a general content database, and organize it into an information pathway	Content formats and categories; gaps in educational resources	The authors identified substantial variation in content and organized resources into three phases—ICU arrival, understanding/partnering in care, and ICU transitions—proposing a digital learning center as a coordinated family education solution.	Contextual resource-development study relevant to digital information delivery for ICU patients and families.	Resource-mapping and gap-analysis study rather than an intervention trial; it does not provide direct evidence on family outcomes or comparative effectiveness.
**Cox et al., 2019 [5]**	PubMed	Effects of a Personalized Web-Based Decision Aid for Surrogate Decision Makers of Patients With Prolonged Mechanical Ventilation: A Randomized Clinical Trial	Multicenter parallel randomized clinical trial	277 patients, 416 surrogates, and 427 clinicians	Adult patients receiving prolonged mechanical ventilation and their surrogates in medical/surgical ICUs	Family-level	Personalized web-based decision aid with prognostic estimates, treatment options, and values clarification	Test whether the decision aid improved clinician-surrogate prognostic concordance versus usual care	Primary: prognostic concordance; Secondary: decisional conflict, psychological distress, communication quality, patient outcomes	The intervention did not improve prognostic concordance or most surrogate/patient outcomes; it produced a modest reduction in decisional conflict but surrogates remained substantially more optimistic than clinicians/prediction model estimates.	Core family/surrogate web-based decision-support intervention in adult ICU care	Conducted in the specific context of prolonged mechanical ventilation; applicability to other ICU surrogate decision-making situations may therefore be limited.
**Das et al., 2019 [15]**	Scopus	Internet health information use by surrogate decision makers of patients admitted to the intensive care unit: a multicentre survey	Multicentre structured survey	473 survey responses from next of kin acting as primary surrogate decision makers	Adult ICU next of kin/surrogate decision makers in 13 Australian ICUs	Family-level	General internet and online health information sources; websites evaluated with HONcode criteria	Investigate use, understanding, trust, and influence of internet and other health information sources among ICU surrogate decision makers	Frequency of online information use; understanding, trust, influence; quality of websites accessed	The ICU nurse and doctor remained the most frequently used information sources, but a substantial proportion also used the internet; internet users reported lower understanding, trust, and influence associated with ICU doctors, while the quality	Direct study of online information-seeking by ICU surrogate decision makers during the ICU stay.	Cross-sectional self-report survey; causal inferences cannot be drawn, and findings may have been influenced by recall and the local health-system context.

										of the 40 websites accessed was moderately high.		
**Hoffmann et al., 2019 [13]**	Scopus	Online information for relatives of critically ill patients: Pilot test of the usability of an ICU families website	Pilot usability test using the Think Aloud method	20 test subjects: 10 lay people and 10 experts (7 nurses and 3 physicians)	Potential ICU relatives and ICU professionals testing a family-facing website	Mixed	Password-protected ICU families website with written explanations, images, videos, forum, and diary function	Pilot test the usability of an ICU families website for relatives of critically ill patients	Usability barriers; task completion; user comments and optimization suggestions	Usability testing identified the main challenges in solving individual tasks and navigating the website; the findings were used to refine the website before larger-scale use.	Early family-facing ICU website usability study directly relevant to digital information support for relatives.	Very small pilot usability sample, including lay people and experts rather than only actual ICU relatives; the study focused on usability rather than effectiveness.
**Pignatiello et al., 2019 [18]**	PubMed	Comparing cognitive load levels among family members of the critically ill exposed to electronic decision aids	Comparative study / cross-sectional analysis from randomized parent trial	97 surrogate decision makers for critically ill patients	Family/surrogate decision makers of critically ill patients	Family-level	Tablet-based electronic decision aids (video-based vs avatar-based)	Compare intrinsic and extraneous cognitive load elicited by two electronic decision aid formats	Intrinsic cognitive load; extraneous cognitive load	Recipients of the video-based decision aid had lower intrinsic and extraneous cognitive load than avatar-based users; after age adjustment, intrinsic-load difference was not significant, while extraneous-load difference remained.	Family-facing electronic decision support tool in critical care decision making	Secondary analytic comparison with a modest sample; it focused on cognitive load rather than downstream family- or patient-centered outcomes.

**Ziyaefard et al., 2019 [33]**	Scopus	Evaluation of the effects of social media-based training on satisfaction and anxiety among the families of patients at the intensive care unit after coronary artery bypass surgery	Interventional study of social media-based family training	Family members of ICU patients after coronary artery bypass graft surgery (exact sample size not extractable from available source snippet)	Family members of adult post-CABG patients admitted to the ICU	Family-level	Social media-based educational/training intervention	Evaluate the effects of social media-based training on family anxiety and satisfaction after CABG-related ICU admission	Family anxiety; family satisfaction	The intervention was associated with reduced anxiety and increased satisfaction among family members of ICU patients after coronary artery bypass surgery.	Relevant primary study on social media-based family education in an adult ICU context, identified in Scopus but not clearly indexed with a PMID.	Single clinical context involving post-CABG ICU admission; limited bibliographic detail outside Scopus and the context-specific sample may reduce generalizability.
**Suen et al., 2020 [6]**	PubMed	Developing the family support tool: An interactive, web-based tool to help families navigate the complexities of surrogate decision making in ICUs	Mixed-methods user-centered development and pilot usability study	6 former ICU surrogates for low-fidelity testing; 14 surrogates and ICU physicians for web-based prototype testing	Surrogates/family members involved in ICU decision making	Mixed	Family Support Tool (interactive web-based support tool)	Develop and pilot-test a scalable web-based tool to support surrogate decision making in ICUs	Usability, acceptability, perceived effectiveness; qualitative feedback	The tool was redesigned to reduce emotional overwhelm and prepare surrogates for family meetings; users judged it highly usable (mean usability score 83.5), acceptable (4.2/5), and effective (4.3/5), and all surrogates said they would recommend it.	Directly develops and tests an ICU family web-support intervention	Development and pilot-usability study with very small testing groups; it was not designed to establish clinical efficacy.
**Petrinec et al., 2021 [20]**	PubMed	Delivering Cognitive Behavioral Therapy for Post-Intensive Care Syndrome-Family via a Mobile Health App	Prospective longitudinal cohort with sequential control/intervention periods	49 family members (24 intervention, 25 control)	Family members of critically ill adult ICU patients at risk of PICS-F	Family-level	Smartphone app delivering cognitive behavioral therapy content	Determine feasibility of app-based CBT delivery for PICS-F	Feasibility, app use, satisfaction, mental health self-efficacy, PICS-F symptoms	App delivery was feasible; family members logged in a mean of 18.6 times and spent a mean of 81.3 minutes using the app; completion rates were acceptable.	Mobile family-support intervention directly targeting PICS-F after adult ICU admission	Small feasibility study using a nonrandomized sequential design; appropriate for feasibility assessment, but limited for drawing efficacy conclusions.

**Shin et al., 2021 [16]**	Scopus	VidaTalk™ patient communication application "opened up" communication between nonvocal ICU patients and their family	Qualitative phase of a mixed-methods study nested within a randomized controlled trial	7 family members participating in 18 semi-structured email interviews	Family members of mechanically ventilated nonvocal adult ICU patients in the intervention group	Family-level	VidaTalk™ patient communication application	Explore family perceptions of the app, communication experience, and emotional reactions while communicating with nonvocal ICU patients	Communication experience; emotions while communicating with the patient	The app opened up clearer and broader family-patient communication; participants described relief, less frustration, and less stress, while some also reported sadness or distress when patients could express worries and anxiety.	Direct family-facing communication app connecting nonvocal adult ICU patients and their relatives.	Very small qualitative sample drawn only from the intervention arm; findings are experience-based and may not generalize broadly.
**Suen et al., 2021 [7]**	Scopus	A Pilot Randomized Trial of an Interactive Web-based Tool to Support Surrogate Decision Makers in the Intensive Care Unit	Pilot randomized trial	Surrogate decision makers and ICU clinicians in a pilot randomized evaluation (exact sample size not fully extractable from available search snippet)	Surrogate decision makers of adult ICU patients and ICU clinicians	Mixed	Interactive web-based tool to support communication and shared decision-making	Assess feasibility, usability, acceptability, and perceived effectiveness of an interactive web-based ICU support tool	Feasibility; usability; acceptability; perceived quality of communication and shared decision-making	The web-based tool was feasible to deploy in ICU settings; surrogates who used the tool reported higher communication and shared decision-making quality scores, although differences versus controls did not reach statistical significance.	Primary trial of a family/surrogate ICU web-based support intervention newly identified through Scopus.	Pilot randomized trial with limited sample size and incomplete detail in the available record; it was likely underpowered to detect between-group differences.
**Rose et al., 2022 [8]**	PubMed	Family perspectives on facilitators and barriers to the set up and conduct of virtual visiting in intensive care during the COVID-19 pandemic: A qualitative interview study	Qualitative descriptive interview study	41 family-member participants	Family members of adult ICU patients who experienced virtual visiting during COVID-19	Family-level	Virtual visiting technologies / video visits	Explore family perspectives on barriers and facilitators to setting up and conducting virtual visits	Thematic barriers/facilitators; family recommendations	Facilitators included preparation, preferred timing, easy-to-use technology, ICU staff presence, and flexibility; barriers included restrictive practices, poor planning, technical issues, and lack of closure at the end of calls.	Direct qualitative evidence on family experience of ICU virtual visiting technologies.	Qualitative study conducted during the COVID-19 period; findings are context-specific and are not intended for statistical generalization.

**Rose et al., 2022 [2]**	PubMed	Psychological distress and morbidity of family members experiencing virtual visiting in intensive care during COVID-19: an observational cohort study	Multicenter prospective observational cohort study	2166 adult family members of ICU patients	Family members of critically ill adult ICU patients using a bespoke virtual visiting solution during COVID-19	Family-level	aTouchAway virtual visiting platform	Evaluate distress, depression, anxiety, and stress among family members experiencing virtual visits	Distress Thermometer; DASS-21; discrete emotions after virtual visits	62% reported severe pre-visit distress; post-visit distress scores decreased by a mean of 1.6 points, although about one fifth of those with repeated visits remained severely distressed; severe-to-extremely severe anxiety or depression affected a notable minority.	Large multicenter study of family psychological outcomes linked to ICU virtual visiting technology.	Observational design without a non-exposed comparison group; distress outcomes were self-reported and may have been shaped by the extraordinary COVID-19 context.
**Valls-Matarín et al., 2022 [35]**	PubMed	Nurse information in Spanish intensive care units: Formal or informal role? Multicentre study	Descriptive cross-sectional multicentre study	228 adult ICUs (brochures collected from 228/280 units)	ICU admission brochures for relatives in adult ICUs	Contextual/resource-based	Brochure-based family information resource (non-digital comparator/context study)	Evaluate the information provided to relatives on ICU admission brochures, especially nursing information	Presence, type, and timing of family information in brochures	All brochures included medical information, but only 21.7% included nursing information; content was generic and heterogeneous, highlighting gaps in formal family-facing information resources.	Broader context study on family information delivery in ICU; useful non-digital comparator for digital resources.	Descriptive study of brochure content rather than a digital intervention study; it does not assess effects on family experience or outcomes.
**Zhou et al., 2022 [17]**	Scopus	Evaluation of the Application Effect of WeChat Platform-based Communication Mode in Family Members of Patients after Partial Pulmonary Resection in the Anesthesia Intensive Care Unit	Comparative evaluation study of a digital communication intervention	Family members of patients after partial pulmonary resection in an anesthesia ICU (exact sample size not fully extractable from available search snippet)	Family members of postoperative adult patients admitted to an anesthesia intensive care unit	Family-level	WeChat platform-based communication mode	Evaluate the effect of a WeChat-based communication mode on anxiety and satisfaction among family members	Family anxiety; satisfaction with medical services; communication-related outcomes	The WeChat-based communication mode was associated with lower anxiety and higher satisfaction with medical services among family members.	Digital communication intervention for adult ICU family members in a postoperative intensive care setting.	Single postoperative anesthesia ICU setting with limited sample detail in the available record; generalizability to broader ICU populations may be limited.

**Haack et al., 2023 [22]**	PubMed	Does an educational website improve psychological outcomes and satisfaction among family members of intensive care unit patients?	Embedded observational analysis within a multicenter cohort	532 family members; 61 accessed the website	Family members of critically ill adult ICU patients in units with flexible visiting hours	Family-level	Educational website about ICU processes and emotions	Evaluate whether website access was associated with better satisfaction and lower anxiety/depression	Family satisfaction (Critical Care Family Needs Inventory); anxiety and depression (HADS)	Website access was associated with better family satisfaction scores and lower prevalence of probable clinical anxiety; no difference was found for depressive symptoms.	Family-focused educational website with measurable ICU family outcomes	Observational comparison with self-selection into website use; because only a minority accessed the website, selection bias cannot be excluded.
**Hoffmann et al., 2023 [3]**	PubMed	Effects of an online information tool on post-traumatic stress disorder in relatives of intensive care unit patients: a multicenter double-blind, randomized, placebo-controlled trial (ICU-Families-Study)	Multicenter double-blind randomized placebo-controlled trial	89 relatives (46 intervention, 43 control)	Relatives of critically ill ICU patients	Family-level	Website with ICU-specific information (vs control website)	Test whether a website with specific ICU information reduced PTSD symptoms and quantify website use	PTSD symptoms (IES); anxiety/depression (HADS); website use	About 50% had clinically relevant PTSD symptoms at baseline; the intervention did not reduce PTSD symptoms or HADS scores versus control, although intervention users generated substantially more website clicks.	Randomized trial of an ICU information website for relatives	Relatively small trial sample and variable intensity of website use; the study may have been underpowered for some psychological outcomes.
**Petrinec et al., 2023 [21]**	PubMed	Self-Care Mental Health App Intervention for Post-Intensive Care Syndrome-Family: A Randomized Pilot Study	Randomized pilot longitudinal study	60 family members (30 intervention, 30 control)	Family members of critically ill adult ICU patients at risk of PICS-F	Family-level	Mental health smartphone app delivering CBT-based self-care content	Test efficacy of smartphone-delivered CBT for reducing PICS-F symptoms	PICS-F symptoms; mental health self-efficacy; quality of life; app use	Anxiety and depression symptom severity decreased over time in the intervention group but not in controls; participants logged into the app a mean of 11.4 times and used it for a mean of 50.2 minutes.	Family-targeted smartphone mental health intervention with pilot efficacy data.	Pilot randomized study with a small sample and preliminary effect estimates; findings would benefit from confirmation in larger confirmatory trials.

**Clarke et al., 2024 [37]**	PubMed	Ethical considerations related to virtual visiting for families and critically ill patients in intensive care: a qualitative descriptive study	Qualitative descriptive interview study	8 newly graduated junior doctors	Clinicians facilitating ICU virtual visits for critically ill patients and families	Professional-facing	Virtual visiting technologies / telehealth for family visitation	Explore anticipated and unanticipated ethical issues raised by ICU virtual visiting	Themes about ethical challenges and benefits	Themes showed virtual visits could support connection and autonomy but also raise concerns around dignity/privacy, emotional distress, and equitable access; the authors recommended virtual communication skills training and accessible family resources.	Contextual qualitative evidence on ethical implementation of ICU virtual visiting for families.	Small qualitative sample based on junior doctors' perspectives; the study lacks direct patient and family data and may not capture wider stakeholder views.
**Riegel et al., 2024 [23]**	PubMed	Family's preferences for and experiences of writing practices in adult intensive care and its use in early bereavement: A descriptive qualitative study	Descriptive qualitative study	16 bereaved participants	Bereaved family members from an adult tertiary ICU	Family-level	Writing practices including ICU diaries, personal journaling, social media, and instant messaging	Explore family preferences and experiences of writing practices during ICU admission and early bereavement	Themes about use/non-use, utility, and bereavement experiences	Writing practices were shaped by prior habits and perceived utility; participants who wrote generally used personal diaries/journals, entries shortened as death approached, and most writings were not reread in early bereavement.	Broader study of digital/non-digital family writing practices connected to ICU communication and memory-making.	Small bereaved sample with retrospective accounts; findings may not transfer to all family members or to earlier phases of the ICU trajectory.
**Xing et al., 2024 [25]**	PubMed	An online delirium detection tool: Cross-cultural adaptation of a Chinese version of the Family Confusion Assessment Method	Cross-sectional validation study	190 critically ill patients and 190 family members	Family members assessing ICU patients for delirium during online visitation	Family-level	Chinese version of FAM-CAM used during online visits	Translate the FAM-CAM into Chinese and test its effectiveness in an online visitation setting	Agreement with ICDSC; sensitivity; specificity; predictive values; AUC	The family-administered online FAM-CAM showed good agreement with nurse ICDSC assessments (kappa 0.759), sensitivity 0.880, specificity 0.890, and AUC 0.881.	Family-facing digital assessment tool used during online ICU visits.	Validation focused on one language and cultural setting and on online visitation; broader clinical or family-centered outcomes were not assessed.

**Zacharelou et al., 2024 [24]**	PubMed	Opinions and priorities for an e-health platform: A member consultation from an intensive care patient organisation	Cross-sectional survey / member consultation	227 participants	ICU survivors and family members connected to a Dutch patient/family organisation	Mixed	Prospective e-health platform for ICU follow-up services	Explore opinions and priorities regarding a future e-health platform for ICU follow-up	Preferences for digital elements, support priorities, confidence in e-health advice	Most participants expressed confidence in advice delivered through an e-health platform; survivors prioritized guidance to relevant professionals, while family members prioritized help with emotional distress.	Broad e-health planning study that highlights digital support priorities of ICU family members.	Consultation on a hypothetical future platform with self-selected members of a patient organisation; the mixed survivor/family sample may limit specificity.
**van Mol et al., 2024 [12]**	PubMed	The usability of a digital diary from the perspectives of intensive care patients' relatives: A pilot study	Cross-sectional online survey pilot study	63 relatives	Relatives of ICU patients using the Post-ICU diary web application	Family-level	Post-ICU digital diary web application	Explore usability of a digital diary from relatives' perspectives	Layout, user friendliness, functionality, relevance, applicability	All but one participant found the diary easy to use and upload photos to; 75% invited other relatives and 61% invited nurses to write; relevance and applicability were rated highly (mean 8.1 and 8.3/10).	Direct family-perspective evaluation of an ICU digital diary tool.	Pilot usability study with self-selected participants; it focused on usability and perceived relevance rather than effectiveness.
**Høyer et al., 2025 [26]**	PubMed	Digital Visits at Intensive Care Units Post-COVID-19: A Mixed-Methods Implementation Evaluation Study	Explanatory sequential mixed-methods implementation evaluation	ICU nurses and managers (exact analytic sample not fully reported in abstract snippet)	Post-COVID ICU digital visit implementation	Professional-facing	Digital video technology for ICU visits	Evaluate use of digital visits in ICUs after COVID-19 and examine implementation from nursing and managerial perspectives	Technology use; perceived support for relationships; implementation needs	About 52.9% of nurses had not used digital visits; users reported support for the patient-relative-nurse relationship, but the technology required reimplementation to fit the post-COVID setting.	Recent implementation study of digital visiting in ICU; included despite staff-oriented lens because it informs sustainability of family visitation technology.	Implementation study with a staff-oriented lens and incomplete sample detail in the available record; direct family outcome data are limited.

**Murray et al., 2025 [9]**	PubMed	User Experience of a Bespoke Videoconferencing System for Web-Based Family Visitation for Patients in an Intensive Care Unit: 1-Year Cross-Sectional Survey of Nursing Staff	Cross-sectional survey of nursing staff	22 ICU nurses	Nursing staff using ICU FamilyLink for web-based family visitation	Professional-facing	ICU FamilyLink bespoke videoconferencing system	Assess nursing staff experience, use scenarios, and future use of a videoconferencing system for family visitation	Modified Telehealth Usability Questionnaire domains; intended future use	Staff reported positive usability across domains; 95% would use the system again when families cannot be physically present and highlighted usefulness for geographically distant relatives.	Implementation/ usability evidence for family visitation technology in ICU, albeit from staff perspective.	Very small cross-sectional staff survey; findings reflect nursing perceptions rather than direct family-reported outcomes.
**Schol et al., 2025 [11]**	PubMed	Determinants of digital ICU diary implementation and use by ICU professionals: A cross-sectional survey analysis	Multicentre cross-sectional survey study	214 ICU professionals	ICU professionals considering implementation of digital diaries offered to patients' relatives	Professional-facing	Digital ICU diary	Identify determinants promoting implementation and utilization of digital ICU diaries	Rated implementation determinants across access, education, offering to relatives, and staff engagement	Top facilitators were seamless accessibility, local champions, and comprehensive education; understanding utility and added value were key for offering diaries to relatives, and 61.7% expressed a positive attitude toward co-writing.	Implementation-focused study relevant to how family-facing digital ICU diaries are introduced in practice.	Professional-facing cross-sectional survey; it is informative for implementation, but does not assess direct family effectiveness or longitudinal outcomes.
**Liu et al., 2026 [32]**	PubMed	Effect of an internet-based, mobile terminal-supported, family-participatory early rehabilitation model on sleep improvement and stigma prevention in critically	Single-center randomized controlled trial	204 adult ICU patients randomized; 196 completed	Adult ICU patients receiving family-participatory rehabilitation guided by a mobile application	Family-level	Internet-based/mobile terminal-supported family-participatory rehabilitation model (IFPER)	Evaluate whether a family-participatory mobile-supported rehabilitation model improved sleep, stigma, and pain	Sleep efficiency and other PSG metrics; stigma; procedural pain	Compared with standard care, the intervention improved sleep efficiency, increased REM sleep, reduced arousals, lowered stigma scores, and reduced procedural pain.	Broader family-participation digital intervention in ICU; patient-centered rather than family-centered outcomes.	Single-center trial with predominantly patient-centered outcomes; the digital component was embedded in a broader rehabilitation model, making attribution

		ill patients: A randomized controlled trial										more cautious.
**Pignatiello et al., 2026 [19]**	PubMed	Evaluation of a Tablet-Based Emotion Regulation Intervention for Surrogate Decision-Makers of Patients With Critical Illness: Pilot Nonrandomized Trial	Pilot nonrandomized trial	48 surrogates (20 usual care, 28 intervention)	Adult surrogates for incapacitated ICU patients (≥48 hours)	Family-level	REFRAME tablet-based emotion regulation app	Evaluate feasibility, acceptability, appropriateness, and preliminary effects of REFRAME on surrogate psychological distress	Implementation outcomes; anxiety; depression	Two-thirds completed all three modules, >70% judged the intervention acceptable/appropriate, and depressive symptoms improved more in the intervention group than in usual care (effect size d=0.68).	Family/surrogate tablet intervention directly targeting acute emotional burden during ICU decision making.	Pilot nonrandomized design with a small sample; preliminary findings remain potentially vulnerable to confounding and would benefit from randomized confirmation.
**Rose et al., 2026 [10]**	PubMed	Implementation and sustainability of an innovative ICU e-diary	Service innovation and evaluation study	380 patients with 1242 diary entries; 35 staff questionnaires	Four ICUs using e-diaries with staff and family engagement	Mixed	ICU e-diary built on aTouchAway	Characterize implementation, family engagement, feasibility, acceptability, and sustainability of an ICU e-diary	Commencement rates; diary entries by staff/family; AIM/FIM feasibility/acceptability; barriers/facilitators	During implementation, monthly e-diary commencement averaged 65%; family members contributed 289 entries; staff described benefits such as accessibility and family participation, while barriers included logins, education needs, and lower technological competence in some families; use persisted over time.	Strong real-world implementation study of a family-contributory ICU e-diary.	Service evaluation and implementation study without a formal control group; real-world results may have been influenced by local workflow and context.

Note. To improve transparency, we added the column “Primary evidence perspective” to distinguish whether the main evidence reported in each study was derived primarily from family members/surrogate decision-makers (Family-level), health care professionals (Professional-facing), both groups (Mixed), or contextual/resource analyses without direct family-reported outcomes (Contextual/resource-based). This classification was assigned according to the primary source of data and perspective represented in each study, rather than according to the overall relevance of the study to the review topic.

Supplementary Table S2. Complete PubMed/MEDLINE search strategy

**Native search string used**
("Caregivers"[Mesh] OR "Family"[Mesh] OR caregiv*[tiab] OR family[tiab] OR families[tiab] OR "care giv*"[tiab] OR carer*[tiab] OR spouse*[tiab] OR "next of kin"[tiab] OR "support person*"[tiab] OR "loved one*"[tiab] OR "significant other*"[tiab] OR partner*[tiab] OR relative*[tiab] OR proxy[tiab] OR proxies[tiab] OR surrogate*[tiab] OR friend*[tiab]) AND ("Intensive Care Units"[Mesh] OR "Critical Care"[Mesh] OR "intensive care unit*"[tiab] OR "intensive care"[tiab] OR ICU[tiab] OR "critical care"[tiab] OR "critically ill"[tiab]) AND ("Social Media"[Mesh] OR "Telemedicine"[Mesh] OR "Mobile Applications"[Mesh] OR internet[tiab] OR online[tiab] OR website*[tiab] OR "web site*"[tiab] OR webpage*[tiab] OR "web page*"[tiab] OR web-based[tiab] OR ehealth[tiab] OR "e-health"[tiab] OR telemedicine[tiab] OR telehealth[tiab] OR videoconferenc*[tiab] OR "virtual visit*"[tiab] OR "virtual visiting"[tiab] OR smartphone*[tiab] OR "mobile app*"[tiab] OR "mobile application*"[tiab] OR app[tiab] OR apps[tiab] OR "social media"[tiab] OR "social network*"[tiab] OR Facebook[tiab] OR Instagram[tiab] OR YouTube[tiab] OR WeChat[tiab] OR WhatsApp[tiab] OR Slack[tiab] OR Messenger[tiab] OR blog*[tiab] OR forum*[tiab] OR podcast*[tiab] OR ChatGPT[tiab] OR "artificial intelligence"[tiab])

Date of search: January–March 2026
Filters applied: last 10 years; Humans; Adult: 19+ years
Note: The PubMed/MEDLINE search string was adapted to the syntax of Scopus and CINAHL while preserving the original conceptual structure of the search.

Abbreviations: Mesh = Medical Subject Headings; tiab = title/abstract.

Supplementary Table S3. Methodological distribution of the 32 included studies

**Primary study design category**	**n**	**%**
Randomized controlled / pilot randomized trials	5	15.6
Nonrandomized interventional / comparative intervention studies	4	12.5
Observational cohort / before-and-after / comparative observational studies	5	15.6
Qualitative studies	4	12.5
Surveys, cross-sectional, consultation, or resource-mapping studies	7	21.9
Usability, development, implementation, or mixed-methods evaluation studies	7	21.9
**Total**	**32**	**100.0**

Studies were grouped according to their **primary methodological design** for descriptive purposes. When a study included multiple methodological components, it was classified according to the design judged to be most central to the study’s aim and reported results.

Supplementary Table S4. Studies reporting limited, null, or mixed effects of digital tools on family-related outcomes

Study	Digital tool category	Outcome assessed	Negative / null / mixed result	Interpretation / note
Hoffmann et al., 2023 [3]	Online information tool / educational website	PTSD symptoms in relatives	No significant reduction in post-traumatic stress symptoms despite use of the intervention website	Suggests that informational tools may improve access and usability but may be insufficient, on their own, to modify more complex psychological outcomes
Cox et al., 2019 [5]	Web-based decision aid	Prognostic concordance; psychological distress; decisional conflict	No clear improvement in prognostic concordance or psychological distress; lower decisional conflict was observed	Indicates selective rather than global effects of decision-support tools
Pignatiello et al., 2019 [18]	Electronic decision aid formats	Cognitive load	Differences in cognitive load varied according to format; effects were not uniformly favorable across all presentation modalities	Suggests that the design and presentation of information may influence user experience, but not always in a straightforward way
Petrinec et al., 2023 [21]	Self-care mental health app	Anxiety and depressive symptoms; feasibility	Preliminary improvement signals, but evidence derived from a small pilot study and remains uncertain	Findings are promising but still exploratory and not sufficient for definitive conclusions
Pignatiello et al., 2026 [19]	Tablet-based emotion regulation intervention	Feasibility; depressive symptoms	Preliminary benefits were reported, but evidence remains limited by pilot nonrandomized design	Suggests possible benefit, but results require confirmation in larger controlled studies
Rose et al., 2022 [2]	Virtual visiting	Distress, anxiety, depression	Virtual visiting was associated with emotional benefit in some families, but high distress levels persisted and the experience could also be burdensome	Highlights the dual nature of virtual visiting: supportive for connection, but emotionally demanding
Clarke et al., 2024 [37]	Virtual visiting / telehealth ethics	Ethical and relational implications	Virtual visiting may mitigate isolation but also intensify ethical tensions related to privacy, dignity, emotional burden, and equity	Shows that benefit depends strongly on governance, mediation, and implementation context
Murray et al., 2025 / Høyer et al., 2025 [9,26]	Video communication systems	User experience; implementation	Systems were generally acceptable, but sustainability depended on staff time, workflow integration, and organizational support	Suggests that usability alone does not guarantee successful long-term implementation
Liu et al., 2026 [32]	Internet-based, family-participatory rehabilitation model	Sleep, stigma, pain	Outcomes were mainly patient-centered rather than family-reported, limiting direct interpretation of family-level benefit	Relevant to family participation, but less informative on family outcomes per se
Digital diaries studies (van Mol 2024; Rose 2026; Schol 2025; Riegel 2024) [10,11,12,23]	Digital diaries / writing practices	Usability, implementation, meaning-making	Benefits were generally positive, but evidence on measurable clinical or psychological outcomes remained limited and context-dependent	Suggests that these tools may have relational and narrative value even when hard outcomes are less clearly demonstrated

Note. This table summarizes studies reporting null, limited, or mixed effects of digital tools on family-related outcomes. The category “negative / null / mixed result” was used descriptively to capture studies in which benefits were absent, inconsistent, selective, preliminary, or strongly dependent on implementation context, rather than to imply overall ineffectiveness of the intervention.
